# The effect of a familiarization critical speed testing session on critical speed determination during treadmill running

**DOI:** 10.1371/journal.pone.0341141

**Published:** 2026-02-09

**Authors:** Lorenzo Micheli, Tommaso Grossi, Silvia Pogliaghi, Francesco Lucertini, Carlo Ferri Marini

**Affiliations:** 1 Department of Biomolecular Sciences – Division of Exercise and Health Sciences, University of Urbino Carlo Bo, Urbino, Italy; 2 Department of Clinical and Experimental Medicine, University of Foggia, Foggia, Italy; 3 Department of Human Movement Sciences, University of Groningen, University Medical Center Groningen, Groningen, The Netherlands; Taichung Veterans General Hospital, TAIWAN

## Abstract

**Background:**

The effect of familiarization with the critical speed (CS) testing process on the outcome of CS tests has yet to be determined.

**Objectives:**

The main aims of the present study were to determine whether a familiarization session prior to CS testing sessions affects time to task-failure (TTF) on subsequent tests and CS estimations, and whether individual characteristics such as sex and fitness status influence any familiarization effect.

**Methods:**

27 healthy adults (10 females, 25 ± 4 yrs) performed the following treadmill protocol: *i)* a maximal incremental test to identify peak speed; *ii)* a familiarization constant-intensity trial (CT_fam_) at the intensity of the first constant-intensity trial used for CS determination (CT_CS_), and *iii)* four randomly ordered maximal constant-intensity trials at ~ 85%, 92%, 98%, and 105% of peak speed. CS was estimated using 2-parameter hyperbolic, linear, and 1/time models including the CT_fam_ (CS not familiarized) or the CT_CS_ (CS familiarized). Familiarized and not-familiarized TTFs and CSs were compared using Wilcoxon tests. Sex effect was analyzed using Mann-Whitney test on the difference in the TTF between CT_CS_ and CT_fam_. The correlation between peak speed and difference in the TTF between CT_CS_ and CT_fam_ was assessed using Spearman’s rank correlation. α was set at 0.05.

**Results:**

CT_fam_ TTF (399 [299] s, median [interquartile range]) was lower (p = 0.009) than CT_CS_ TTF (495[316] s), whereas CS familiarized and not-familiarized were not different in any model (p > 0.05). Sex did not affect the differences between familiarized and not-familiarized TTFs and CS. However, differences in the TTF between CT_CS_ and CT_fam_ were negatively correlated with peak speed (ρ = −0.381, p = 0.050).

**Conclusions:**

Familiarization with constant-intensity trials affected the TTF but not CS. Importantly, the familiarization effect was larger in less fit individuals, showing a negative correlation between the TTF differences (i.e., CT_CS_ minus CT_fam_) and peak speed.

## Introduction

The maximal metabolic steady-state (MMSS) is a key determinant of exercise capacity that is crucial for effective aerobic exercise prescription and prediction of physical performance [1]. The MMSS represents an exercise intensity boundary that separates exercise work rates that can be sustained under homeostatic conditions from those that cannot [2]. Therefore, accurate determination of this threshold is crucial for ensuring effective exercise prescription and training that will improve the chances of enhancing both performance and health. In this regard, recent studies attempted to address the main challenges associated with running exercise intensity prescription [3–5].

One of the strategies used to assess the MMSS is critical power (CP) or its analogous critical speed (CS) [6]. The CS concept is based on the relationship between exercise intensity and the duration over which a specific task (e.g., running, cycling, rowing, swimming) can be sustained. Although there is no consensus on the exact standardized protocol needed to determine these thresholds [7,8], they are usually determined using 3–5 constant-intensity trials that elicit a time to task failure (TTF) between 2–20 min and are conducted on separate days [9,10]. Plotting TTF against power output, or speed, results in a hyperbolic relationship that provides an asymptote that gives the CS.

Several factors can influence the estimation of this boundary from a given power-time series dataset, including the duration of predictive trials included in the model fit [7,11], the cycling cadence [7,12], and the number and duration of the included trials [11]. Another crucial factor for accurately determining CS is the requirement that each constant-intensity trial is performed at maximal effort and to exhaustion. Indeed, even slight differences in TTF from a constant-intensity trial have been shown to lead to significant variations in CP estimation [11,13,14]. In this respect, participants' familiarity with a given task has been shown to significantly affect the results of field [15] or laboratory [16] tests used to determine CS, as well as sport performance [17]. For example, Galbraith et al. [15] proposed that changes in CS following familiarization (as evidenced by a smaller coefficient of variation [CV] of the CS parameter) might result from improved pacing strategies during repeated time trials. In laboratory conditions, Triska et al. [16] showed that a familiarization of cycling time trials performed for 12, 7, and 3 min improved average power output resulting in a slightly higher but not significantly different CP estimate compared to the unfamiliarized condition. A lower standard error of the estimate (approximately 30% for the CP parameter) was also reported when using the time trials after familiarization, indicating increased accuracy and the presence of a learning effect. However, these results were based on tests involving time trials with fixed durations, where the pacing strategy plays a significant role. In contrast, pacing strategies are not a concern during TTF trials, as the pace is dictated by a fixed treadmill speed or bike wattage, eliminating the variability introduced by self-selected pacing. Therefore, although conducting a familiarization session may enhance the accuracy of CP or CS estimates in time trial conditions [15,16], to our knowledge, no studies have investigated if similar effects would be observed when using constant-intensity TTF protocols, which involve trials having fixed treadmill speed or bike wattage. Therefore, this study aimed to investigate whether conducting a familiarization session of a TTF trial before constant-intensity trials used for determining CS affects TTF during constant-intensity trials and, consequently, CS estimation. The hypothesis of the present study is that performing a familiarization session increases the TTF and consequently affects the accuracy of the CS estimates. Additionally, we hypothesized that the familiarization effect may be associated with individual characteristics such as fitness level and sex.

## Methods

### Participants

After providing written informed consent, 27 healthy adults (17 males, age: 25.1 ± 3.7 yrs; height: 1.7 ± 0.1 m; body mass: 72.2 ± 6.1 kg; body fat [BF] 7.0 ± 1.8% and 10 females, age: 23.7 ± 3.5 yrs; height: 1.6 ± 0.1 m; body mass: 56.2 ± 7.0 kg; BF 17.8 ± 3.6% [mean±SD]) voluntarily participated in this study. Participants were physically active, engaging in at least 30 minutes of moderate-intensity physical activity at least 3 days a week in the last 3 months before enrollment, with no experience in laboratory testing. All participants had no history of any cardiovascular or respiratory diseases or musculoskeletal injuries and were free of any medical conditions or treatments that could affect metabolic and cardiorespiratory responses to exercise. This study was reviewed and approved by the Institutional Research Ethics Board on June 29, 2023. Participant recruitment was conducted from July 10, 2023, to June 30, 2024 (Reference number: RN71–29062023).

### Sample size

The sample size was *a priori* calculated with the aim of verifying the differences in TTF and CS between the familiarized and unfamiliarized conditions. A total of 27 participants resulted required to achieve a statistical power of 0.80 with α equal to 0.05 using a two-tail paired sample *t*-test with a Cohen's effect size of 0.556. Since this is the first study assessing the effect of familiarization, the estimated effect size was calculated based on the results of a test-retest study [18].

### Experimental design

Participants visited the laboratory on 6–7 occasions, with each visit separated by at least 48 hours and completed the protocol in about 4 weeks. Participants were instructed to maintain their usual exercise regimen and dietary habits, while avoiding vigorous physical activity, and alcohol and caffeine intake on the day of testing. Additionally, they were asked to stay well-hydrated the day before testing, consume 0.5 liters of water one hour prior to their scheduled sessions, and arrive at the laboratory after fasting for at least three hours. Compliance with these instructions was assessed before each testing session, and tests were postponed if any instructions were not adhered to. All tests were conducted in the same laboratory by running on a motorized treadmill (Runrace model, Technogym, Cesena, Italy), which was calibrated according to the manufacturer's instructions. The laboratory environment was maintained at a temperature ranging between 20–24°C and a relative humidity of 40–50%.

On Day 1, the highest speed reached (SP_peak_) was measured during a maximal incremental test. Participants were not aware of the study aims and thus did not know that the intent of the session on Day 2 was to familiarize them with a TTF trial. On Day 2, participants performed a familiarization session using the same procedures and exercise intensity that was used on Day 3. Exercise intensities of the constant-intensity trials from Days 3–6 were randomly selected as 85%, 92%, 98%, and 105% of the SP_peak_ achieved during the maximal incremental test on Day 1. Additional CTs were performed if certain criteria were not met (see Constant-intensity trials paragraph).

The participants were kept blind about the order of the sessions they were going to perform. Therefore, the treadmill display was covered to ensure participants remained unaware of the exercise intensity during the CTs (Days 2–6). The treadmill slope was fixed at 0% during all the tests.

### Assessments

#### Anthropometry and maximal values (Day 1).

On Day 1, anthropometric measurements including body mass (barefoot, at nearest 0.5 kg), height (barefoot and head in the Frankfurt plane, at nearest 0.01 m), and skinfold thickness (chest, abdomen, and thigh sites in men, and abdominal, triceps, chest, midaxillary, subscapular, suprailiac, and thigh sites in women, at the nearest 0.001 m), were taken. Skinfold thickness was measured in triplicate and BF percentage was calculated according to the American College of Sports Medicine’s (ACSM) guidelines [19].

Then, after assessing pre-exercise values, participants performed a 3-minute warm-up at 40% of their estimated (as described below) maximal intensity followed by the maximal incremental test, using a customized running ramp protocol [20]. Briefly, *V̇*O_2max_ was estimated using a model proposed by Matthew et al. [21], which is based on a questionnaire validated in different populations [22,23] compiled prior to the maximal incremental test. The speed expected to elicit the estimated *V̇*O_2max_, was calculated using the metabolic equations published by ACSM [19] and was set as the “final” stage of the maximal incremental test. The initial speed of the ramp protocol was set to 50% of the estimated final stage speed. Thereafter, the speed of the treadmill was adjusted every minute with fixed increments determined for each participant by dividing the difference between the final and initial speeds by 10 minutes, then multiplying by the number of minutes elapsed from the start of the test (excluding warm-up) to the beginning of each stage. This approach was designed to ensure that the estimated *V̇*O_2max_ would be reached around the 10^th^ minute of the test [24], which represents a midway between 8 and 12 minutes, the currently recommended duration for a maximal incremental test [25].

During maximal incremental test, participants were verbally encouraged to continue until task failure, which was determined in the moment when participants step off the treadmill or asked to stop the test. The SP_peak_ was then computed as described in the Data analysis section.

#### Familiarization session (Day 2).

On day 2, a familiarization session was performed. This session was carried out to task-failure using the same protocol and exercise intensity of the constant-intensity trial that was performed on day 3 (CT_CS_) (see Constant-intensity trials), which allowed participants to familiarize with the specific exercise maximal effort they would experience in the next session.

#### Constant-intensity trials (Day 3–6).

During the CTs days, participants performed, in randomized, counterbalanced order, constant load exhaustive exercise sessions at 85%, 92%, 98%, and 105% of SP_peak_. The sessions began with a 4-minute warm-up: 2 minutes at the intensity corresponding to 40% of SP_peak_ and 2 minutes at 50% of SP_peak_. After the warm-up, the participants were asked to jump off the treadmill by placing their feet on the side support and, in the meantime, the treadmill belt speed was increased to the desired speed. At the end of this transition period, which was standardized to 60 seconds, the participants jumped back onto the treadmill and started again to run. The participant was asked to maintain this intensity as long as possible until task failure and was verbally encouraged as task failure approached. TTF was measured from the moment the participant started running at the target speed, until the participant was no longer able to maintain the intensity, determined as the moment when the participants placed their hands on the side supports, jumped off the treadmill on the side steps, or verbally asked to stop the test. Each participant initially performed 4 CTs. However, additional trials were performed if the CV of the estimated CS exceeded 5% in every model (see the Data analysis paragraph) in the CS parameter [18]. For safety reasons, a chest harness was worn by participants during each test.

### Data analysis

#### Peak speed.

SP_peak_ was defined using the following formula [26]:


$${\text{SP}}_{\text{peak}}={\text{V}}_{\text{completed}}+\text{t}/\text{TSD}\;\text{x}\;\Delta \text{s}$$

where V_completed_ corresponds to the running speed of the last fully completed increment, t is the time spent in the following uncompleted increment, TSD is the total step duration, and Δs is the running speed increment between the last two stages.

#### Critical speed.

CS was determined using the following 3 models by means of the Critical Power web app (https://www.exphyslab.com/cp):

2-parameter hyperbolic model (CS_2-hyp_):


$$\text{TTF}={\text{D}}^{\prime }/(\text{SP}-\text{CS})$$

linear model (CS_lin_):


$${\text{D}}_{\text{lim}}={\text{D}}^{\prime }+\text{CS}\;\text{x}\;\text{TTF}$$

1/time linear model (CS_1/Tlim_):


$$\text{SP}={\text{D}}^{\prime }\;\text{x}\;(\text{1}/\text{TTF})+\text{CS}$$

where CS is critical speed, D’ is the fixed distance that can be sustained above CS expressed in meters, D the running distance (e.g., speed x time) in each constant-intensity trial expressed in meters, TTF the time to task failure corresponding to each CT, and SP the speed sustained during the trial.

The TTF collected during the CTs were used to obtain CS as follows:

- not familiarized CS (CS_notfam_): using the TTFs of the constant-intensity trial performed on day 4–6 and day 2 (CT_fam_);
- familiarized CS (CS_fam_): using the TTFs of the constant-intensity trial performed during day 4–6 and day 3 (CT_CS_).

Additionally, CS_notfam_ and CS_fam_ model reporting the best fit in each participant was determined as the model exhibiting the smallest standard error of the estimate in the CS parameter. Then, the CS estimated using the three models (i.e., 2-parameter hyperbolic model, 2-parameter linear model, 2-parameter 1/time linear model) and the best fit were used in the statistical analysis.

### Computer simulation

To assess the hypothesis that an underestimation error in the TTF affects CS estimation differently based on the intensity of the constant-intensity trial, a computer simulation was conducted using data collected in the present study.

The TTF values of the constant-intensity trial performed in sessions 3–6 were used to simulate four scenarios with a change of the TTF of 5% (i.e., actual TTF in sec multiplied by 1.05) in either the CT with low, mid-low, mid-high, or high intensity. Then, the CS were estimated using the actual and the simulated TTF values across different CT intensities.

### Statistical analysis

Data are presented as mean ± SD or median [interquartile range]. The following analyses were performed using JASP software (version 0.18.0) to evaluate the effect of a familiarization session on TTF and on the derived CS. Due to violation of the normality assumption, as indicated by the Shapiro-Wilk test, non-parametric analyses were performed.. Specifically, TTF from CT_fam_ and CT_CS_ and CS_notfam_ and CS_fam_ were compared by means of Wilcoxon tests and their effect size was reported as the rank biserial correlation (r_rb_). The effect of sex on the differences in TTF between CT_CS_ and CT_fam_ (ΔTTF) and the differences between CS_notfam_ and CS_fam_ (determined using a 2-parameter hyperbolic model, linear model, 1/time linear model) was analyzed using the Mann-Whitney test. Correlation between fitness status expressed as SP_peak_ and the difference in the ΔTTF was assessed by means of Spearman's rank correlation. The familiarization effect, expressed as the difference between CS_notfam_ and CS_fam_, was compared among the models used to determine CS (i.e., 2-parameter hyperbolic model, linear model, 1/time linear model) using a Friedman test. α was set at 0.05.

## Results

### Descriptive results

Out of the 27 participants, CS could be determined in 23 participants because four participants did not obtain an accurate CS estimation model (i.e., CV of the estimated CS parameter above 5%), as specified in the *Constant-intensity trials* paragraph. The SP_peak_ reached during the maximal incremental test was 17.0 ± 1.5 km·h^-1^ and 13.1 ± 1.5 km·h^-1^ for males and females, respectively.

### Differences in TTF between CT_CS_ and CT_fam_ and between CS_notfam_ and CS_fam_

**Fig 1:**
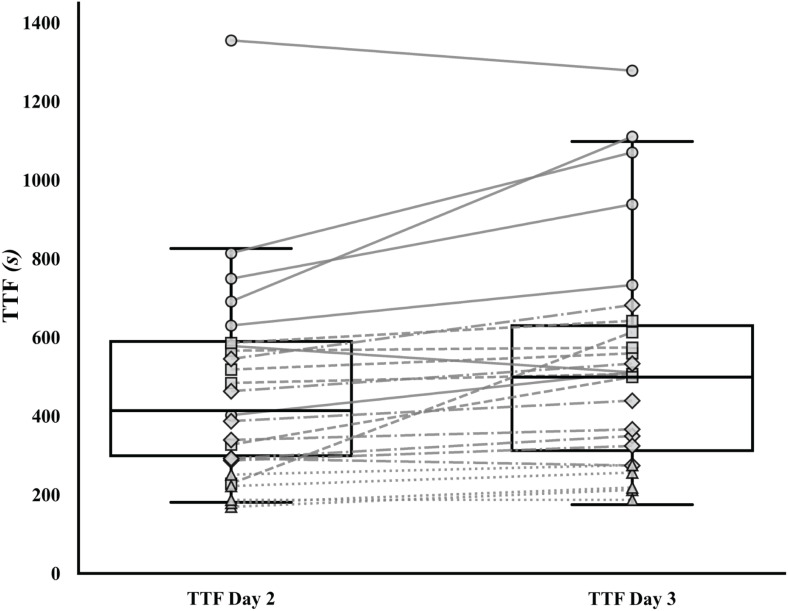
Familiarization effect on CS across different familiarization intensities. Box plot representing individual performance expressed as the TTF between Day 2 and 3. TTF, time to task failure, Familiarization intensities: Grey circles, low intensity (~ 85% of peak speed); Grey squares mid-low intensity (~ 92% of peak speed); Grey diamonds, mid-high intensity (~ 98% of peak speed); Grey triangles, high intensity (~ 98% of peak speed).

The TTF at CT_CS_ (495 [316] s) was higher (p = 0.009, *r*_*rb*_ = 0.593) than TTF at CT_fam_ (399 [299] s) resulting in a median value of Δ_TTF_ of 19 [81] s (Fig 1). The CS_notfam_ was not different from CS_fam_ when determined using 2-hyperbolic (p = 0.629), linear (p = 0.732), and 1/time (p = 0.695) models or best fit approach (p = 0.810). A detailed report of the CSs determined with each model is shown in Table 1, (raw individual data can be found in S1 File).

**Table 1:** Median and [IQR] of CS_notfam_ and CS_fam_ determined utilizing each mathematical model (n = 23).

Model	CS_notfam_ (km·h^-1^)	CS_fam_ (km·h^-1^)	ΔCS (km·h^-1^)
2-hyp	11.5 [2.4]	11.4 [2.2]	0.0 [0.1]
linear	11.7 [2.1]	11.8 [2.0]	0.0 [0.1]
1/time	12.1 [2.2]	12.3 [2.1]	0.0 [0.2]
best fit	11.5 [2.1]	11.4 [2.1]	0.0 [0.1]

IQR, range interquartile; CS_notfam_, critical speed not familiarized; CS_fam_, critical speed familiarized; ΔCS, difference between CS_notfam_ and CS_fam_; n, number of participants; ΔCS, differences between CS_notfam_ and CS_fam_; 2-hyp, two parameters hyperbolic model, 1/time, 1/time linear model.

The familiarization effect, expressed as Δ_TTF_ (p = 0.052) and differences between CS_notfam_ and CS_fam_ determined using the 2-hyperbolic (p = 0.728), linear (p = 0.950), 1/time (p = 0.313), and best fit approach (p = 0.633), were not affected by sex. Δ_TTF_ showed a significant (p = 0.050), negative (ρ=−0.381) correlation with SP_peak_. On the other hand, no significant correlations were found between the difference between CS_notfam_ and CS_fam_ determined by using the best fit approach and SP_peak_ (p = 0.998), showing a negligible negative relationship (ρ = 0.000). The familiarization effect, expressed as the difference between CS_notfam_ and CS_fam_, was not affected by the CS estimation model (χ^2^_(3)_= 2.968; W = 0.043 p = 0.397).

**Fig 2:**
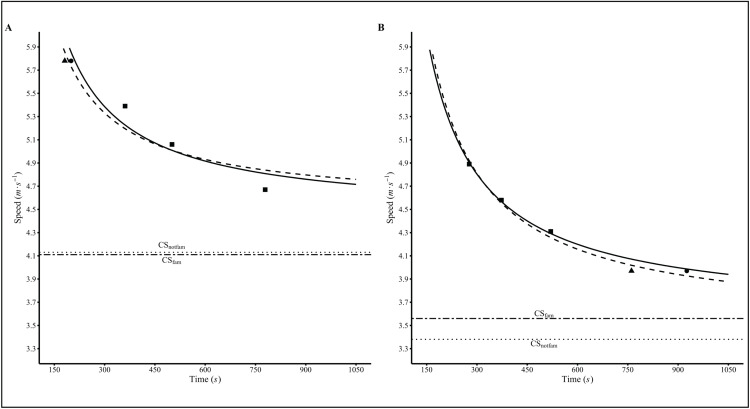
Critical speed plots of two representative participants performing high and low familiarization trial intensity. Individual TTF and CS data of two representative participants who performed the familiarization session at high (panel A) and low (panel B) intensity, respectively. TTF, time to task failure; CS, critical speed; Triangle, TTF and speed data from Day 2; Circle, TTF and speed data from Day 3; Squares, TTF and speed data from Day 4-6; CS_fam_, critical speed obtained combining data from Days 3-6; CS_notfam_, critical speed obtained combining data of Day 2 and Days 4-6).

The effect of the exercise intensity (i.e., %SP_peak_) utilized during the familiarization session on the TTFs and CSs estimation of each model are descriptively reported in Table 2. Moreover, a graphical representation of how the speed-time relationship is affected by the exercise intensity utilized for familiarization is displayed in Fig 2.

**Table 2:** Mean±SD of the descriptive results regarding the effect of the exercise intensity of the familiarization session on the differences between TTFs and CS_notfam_ and CS_fam_.

Familiarization intensity	Speed (km·h^-1^)	ΔTTF (s)	ΔCS_2-hyp_ (km·h^-1^)	ΔCS_lin_ (km·h^-1^)	ΔCS_1/time_ (km·h^-1^)	ΔCS_bestfit_ (km·h^-1^)
Low	12.6 ± 2.1	109.1 ± 175.9	−0.2 ± 0.6	−0.1 ± 0.4	−0.1 ± 0.2	−0.1 ± 0.5
Mid-low	13.8 ± 1.7	90.2 ± 144.9	0.2 ± 0.3	0.1 ± 0.1	0.0 ± 0.1	0.1 ± 0.1
Mid-high	15.8 ± 3.0	27.7 ± 47.1	0.0 ± 0.1	0.1 ± 0.1	0.1 ± 0.2	0.0 ± 0.1
High	16.5 ± 2.6	4.0 ± 17.2	0.0 ± 0.1	0.0 ± 0.1	0.1 ± 0.2	0.0 ± 0.1

CS_notfam_, critical speed not familiarized; CS_fam_, critical speed familiarized; ΔCS_2-hyp_, differences in CS_notfam_ and CS_fam_ determined using the 2 parameters hyperbolic model; ΔCS_lin_, differences in CS_notfam_ and CS_fam_ determined using the linear model; ΔCS_1/time_, differences in CS_notfam_ and CS_fam_ determined using the 1/time model; ΔCS_bestfit_, differences in CS_notfam_ and CS_fam_ determined using the best fit model; Low, intensity corresponding to ~ 85% of peak speed (number of participants [n]: CS = 6; TTF = 7); Mid-low; intensity corresponding to ~ 92% of peak speed (n: CS = 5; TTF = 6); Mid-high, intensity corresponding to ~ 98% of peak speed (n: CS = 5; TTF = 7); High, intensity corresponding to ~ 105% of peak speed (n: CS = 5; TTF = 5).

**Fig 3:**
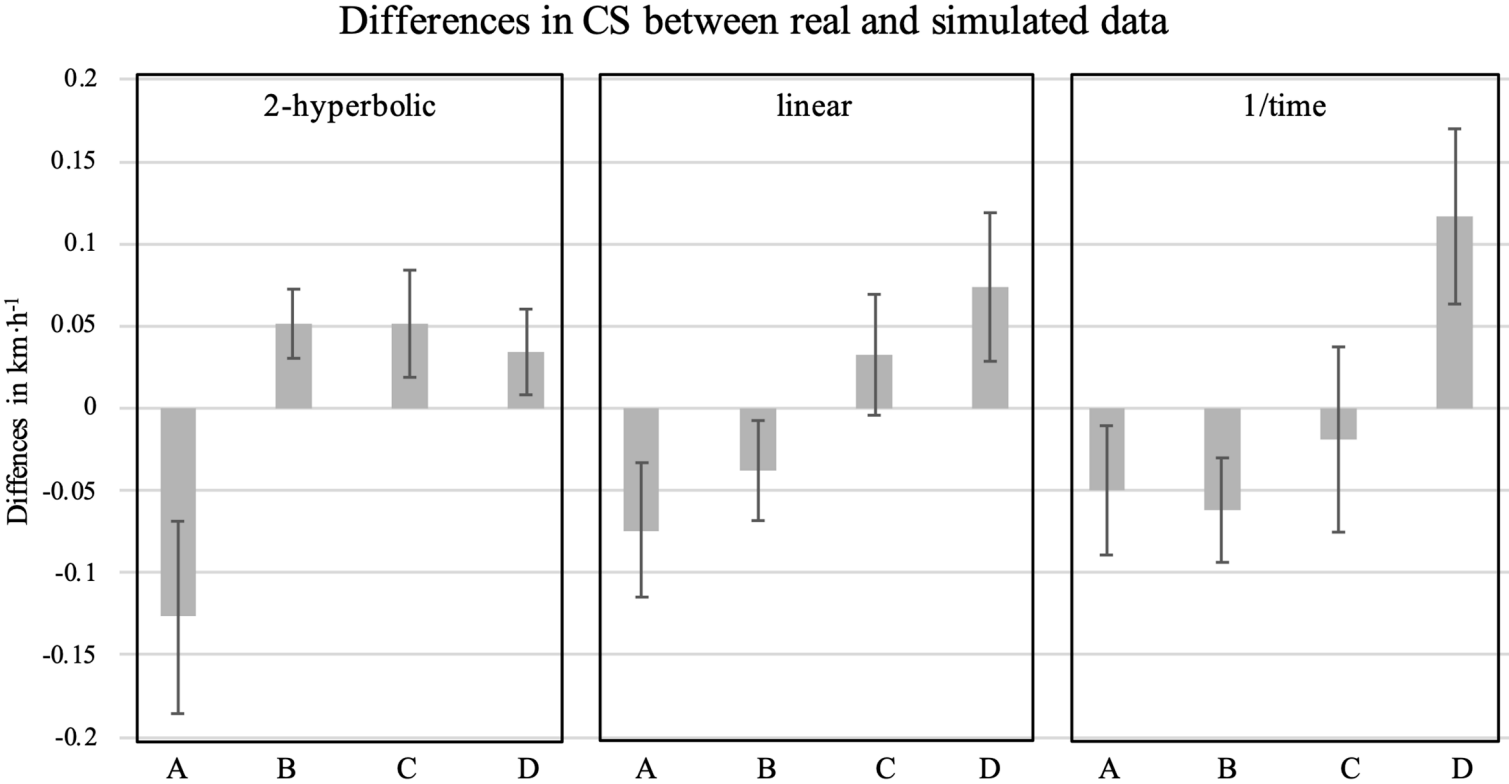
Differences between CS estimates derived from real and simulated data across different mathematical models. CS, critical speed; A, difference between CS determined using real data and CS determined using simulated data with a 5% higher TTF at low intensity (~85% of peak speed); B, difference between CS determined using real data and CS determined using simulated data with a 5% higher TTF at mid-low intensity (~92% of peak speed); C, difference between CS determined using real data and CS determined using simulated data with a 5% higher TTF at mid-high intensity (~98% of peak speed); D, difference between CS determined using real data and CS determined using simulated data with a 5% higher TTF at high intensity (~105% of peak speed).

As regard the computer simulation, the mean and SD of the actual and underestimated CS at each CT intensity are reported in Table 3 and a graphical representation is available in Fig 3.

**Table 3:** Mean and SD of the CS determined using both the actual and simulated TTF.

Model	Intensity				
	Real	Low	Mid-low	Mid-high	High
2-hyperbolic	11.6 ± 1.8	11.7 ± 1.8	11.5 ± 1.8	11.5 ± 1.8	11.5 ± 1.8
linear	11.8 ± 1.9	11.9 ± 1.9	11.9 ± 1.9	11.8 ± 1.9	11.8 ± 1.9
1/time	12.1 ± 2.0	12.2 ± 2.0	12.2 ± 2.0	12.2 ± 2.0	12.0 ± 2.0

SD, standard deviation; CS, critical speed; TTF, time to task failure; Low, CS determined using 5% higher TTF corresponding to the intensity of ~ 85% of peak speed; Mid-low, CS determined using 5% higher TTF corresponding to the intensity of ~ 92% of peak speed; Mid-high, CS determined using 5% higher TTF corresponding to the intensity of ~ 98% of peak speed; High, CS determined using 5% higher TTF corresponding to the intensity of ~ 105% of peak speed.

## Discussion

We evaluated the effect of familiarization on the estimation of CS in treadmill running. Our data demonstrated that the TTF at the same running speed (i.e., resulting from CT_fam_ and CT_CS_) is significantly increased after familiarization. This outcome seems to suggest the presence of a learning effect during high-intensity exhaustive running exercise and fits within the findings from previous studies [15,27] on other types of tests. However, although differences in TTF were observed, these differences did not translate into variations in CS. This somewhat contradicts expectations and contrasts with previous studies which, attempting to investigate how the combination of various trials (e.g., the shortest, the longest etc.) affects the final estimate of CP, reported significant differences in CP with even minor changes in TTF [11,13,14]. Moreover, the correlation analysis between fitness status (as assessed by SP_peak_) and Δ_TTF_ demonstrated smaller changes in TTF in participants with greater SP_peak_, suggesting that fitter people benefit less from a familiarization session of high intensity running than less fit individuals. This outcome also entails that the participants less accustomed to high-intensity or maximal exercises, who usually are represented by less trained individuals, may need a familiarization session before performing CS tests. In addition, although the effect of sex on the Δ_TTF_ was not statistically significant, there seems to be a possible greater familiarization effect in females when compared to males.

Previous studies reported that the model used to estimate CP or CS plays a key role in its determination [7,11]. However, the results of this study do not align with the above-mentioned previous research and indicate that there were no differences in the effect of the familiarization session among the models used, with no model appearing to be more robust or affected by the presence of the familiarization session more than the others.

Furthermore, from the data presented in the present article, it appears that the session order (i.e., the intensity at which the familiarization session is carried out) could have an influence on the subsequent determination of CS. Although the authors primarily explored this aspect descriptively, a noticeable pattern emerged in the differences between CS_notfam_ and CS_fam_ as determined by each model. Notably, performing the familiarization session at the lowest intensity (i.e., ~ 85% of SP_peak_) may result in a trend opposite to the others, leading to an underestimation of CS_notfam_ compared to CS_fam_. To further analyze this aspect, a computer simulation has been performed, which confirmed that errors in TTF estimation (i.e., higher actual TTF) differentially affect CS estimation depending on the intensity of constant-intensity trials, with an increase of the TTF of the longest (lowest-intensity) trial leads to a higher CS estimate, whereas increasing the TTF of the shortest (highest-intensity) trial results in a lower CS estimate. This highlights how familiarization-related TTF errors can systematically bias CS, depending on which trial is used as first intensity. Thus, the results of this study, along with the computer simulation, suggest that the randomization of the constant-intensity trial intensity order may have attenuated the familiarization effect, which was otherwise evident in the TTF. These findings highlight that TTF underestimation errors could differentially affect CS estimation based on constant-intensity trials intensity. Therefore, to ensure reliable experimental outcomes and effective application in performance monitoring and training prescription, careful and standardized structuring of trial order and intensity seems to be essential to minimize errors and ensure reproducibility. This aspect, especially if it will be confirmed by future studies, could raise an additional issue regarding the methodology for determining CS. In fact, the choice of the order of the exhaustive trials and their randomization, which is often poorly reported in the literature, could affect the CS test results and their interpretation and should be always included for reducing the possible biases due to arbitrary choices made in the protocol selection. Based on the findings of this study, which highlighted that the trials at the extremes (i.e., the longest and the shortest) may have the greatest influence on CS estimation, the authors recommend, in the absence of randomization, of conducting the first trial or a familiarization session at the mid-intensity trials, since they seem to have the smallest leverage effect on CS estimation. This approach will also help practitioners to ensure and possibly adapt the intensity of the highest and lowest trials based on the results of the first trial to generate an even distribution of the TTF. Notwithstanding, recognizing that additional familiarization sessions may not always be practical, it is noteworthy to highlight that when the CS criteria (e.g., CV below 5%) are respected and a proper randomization has been performed, the familiarization session might not be needed.

Because the number of observations was limited (i.e., CS data from 23 participants), it was only possible to assess the familiarization effect at different constant-intensity trial intensities used for the familiarization trials by means of descriptive statistics and a computer simulation, which represents a potential limitation of the present study. Although partially explored with the above-mentioned computer simulation, we could not determine the optimal intensity at which familiarization should be performed. In this regard, future studies should aim to analyze the effect of session order, as well as whether familiarizing participants at each testing intensity provides more robust CS estimations. This will help identify whether certain intensities require more familiarization or have a larger impact on CS estimations more than others. Finally, future studies should evaluate if including familiarization sessions performed at submaximal CT not to failure, may represent a practical strategy to improve CS estimation and help participants become accustomed to all testing intensities without increasing the overall testing burden.

On the other hand, a limitation of this study is that we did not confirm whether the CS accurately corresponded to the MMSS by measuring oxygen consumption or blood lactate concentration during a prolonged run. Therefore, it is not possible to determine whether one CS (e.g., CS_notfam_ or CS_fam_) is superior to the other at individual level, and especially whether this may depend on the intensity at which familiarization was performed.

## Conclusion

In conclusion, the present study highlighted that a familiarization session could be useful if the main outcome is represented by the TTF during maximal running exercises, especially in less fit individuals who are less accustomed to maximal effort. In addition, findings suggest that, rather than randomization, there may be an optimal order for completing exhaustive trials in the construction of the speed-time relationship. Elucidation of this concept could reduce variability among studies and improve reproducibility of CS measurement.

## Supporting information

S1 File Participants individual raw data of both CS and TTF. CS, Critical Speed; TTF, Time to Task Failure.
(XLSX)
